# ‘When I asked for help and support it was not there’: current NHS employment practice and its impact on people with systemic lupus erythematosus

**DOI:** 10.1093/rap/rkab019

**Published:** 2021-03-12

**Authors:** Sara Booth, Elizabeth Price, Elizabeth Walker

**Affiliations:** 1 Cambridge Breathlessness Intervention Service, University of Cambridge, Cambridge; 2 Faculty of Health Science, University of Hull, Hull, UK

**Keywords:** SLE, employment, National Health Service, vocational rehabilitation, occupational health, poverty, flexibility, fatigue

## Abstract

**Objectives:**

The aim was to investigate whether National Health Service (NHS) employees with SLE, for whom work disability and early retirement are high, are supported effectively in at work.

**Methods:**

An online survey of 393 people with lupus was completed through the LUPUS UK website, investigating participants’ experiences in maintaining employment. Quantitative and qualitative data were collected. Disease fluctuation, invisibility and fatigue were identified as having substantial negative impacts on employment. This study examined data from a large subgroup (*n* = 72, 18.74%) of current/previous NHS employees. Descriptive statistics and thematic analysis were used to explore and characterize the demography and experiences of participants.

**Results:**

The NHS subgroup (*n* = 72) represented 18.74% of the whole cohort; 100% were female and of working age (18–64 years). Fifty-one were currently (70.8%) and 21 previously (29.2%) NHS employees. Forty-nine (60%) were clinicians. Twenty-one (29.16%) of this working-age subgroup had left any employment. Negative effects of SLE on employment were universal (including an impact on career choices, work disability, enforced part-time working, lower income and early retirement). NHS support for participants to maintain employment was inconsistent, with more negative experiences than positive. The impact of SLE on employment seemed to be poorly understood.

**Conclusion:**

A punitive approach to NHS employees with SLE was more common than a proactive, flexible, problem-solving one despite inclusive rhetoric, resulting in the loss of skills and experience to the service. Characterizing conditions such as SLE and long coronavirus disease 2019 as fluctuating, invisible conditions with constitutional symptoms highlights features with negative employment impact, potentially facilitating much-needed change in NHS organizations, with greater use of occupational health, vocational rehabilitation, redeployment and retraining opportunities, highlighting the need for evidence-based employment interventions and improved management of fatigue.

Key messages

- Fluctuation, invisibility and fatigue are recognized as having detrimental effects on people with SLE maintaining employment.
- National Health Service employees with SLE retire early, experience work disability and are not supported by National Health Service employment practices.
- Characterizing the negative employment impacts of SLE as a fluctuating invisible condition with constitutional symptoms might improve employment support and research.

## Introduction

The National Health Service (NHS) is a widely admired, generally cost-effective, free at the point of need, taxpayer-funded health-care provider for the UK. Its remit is to ‘to improve … health and wellbeing, supporting us to keep mentally and physically well, to get better when we are ill and, when we cannot fully recover, to stay as well as we can … care and compassion … matter most’ [1].

The NHS is the fifth largest global employer, and the largest in the UK, with a workforce of 1.3 million [2]. It has a long-standing skills shortage, with difficulty recruiting and retaining staff, in both clinical and non-clinical roles [3]. The recent NHS People Plan [3] states that staff recruitment and retention are priorities that will be achieved, in part, by improving the work experience. The plan recognizes that many people feel overstretched and undervalued and that workforce diversity, including the employment of more people with long-term conditions and disabilities (LTC/Ds), need to be extended [3, 4]. The skills shortage has been highlighted and exacerbated by the coronavirus disease 2019 (COVID-19) pandemic.

Employing more people with LTC/Ds has been a stated aim of the NHS for some time. A Department of Health report published 20 years ago [5] stated that NHS ‘employers need to develop and sustain good equal opportunities practices, which will enable disabled people to gain NHS jobs and to retain their active mainstream employment…’ because NHS ‘employers are missing out on a huge pool of talent’. Around the same time, the ‘Improving Working Lives’ [6] report pledged that the NHS would give employees greater opportunities to work flexibly.

This paper focuses on the experiences of NHS employees with SLE (lupus) a currently incurable, multisystem disease, with a fluctuating, unpredictable course, which ‘commonly results in debilitating chronic ill-health’ [6]. Its onset in youth, its incurability and debilitating nature over many years can have profoundly negative consequences for the education, employment opportunities and life chances, in addition to the quality of life, of many people diagnosed and living with lupus [7–9].

Unemployment figures for people with lupus have been cited variously as 59% [10], 49.78% [11] and 57.1% [12]. The potential number of working years lost for each individual (and the disease group) is relatively large [7, 10–12].

In addition, given that lupus affects more women than men (ratio 9:1), is more prevalent in those with Afro-Caribbean or East Asian heritage and has a peak onset in ‘young women between the late teens and early 40s’ [7–9], the inter-sectionality of workplace disadvantage might be a significant factor in the employment experiences of people living with lupus.

The impacts of unemployment, including poverty, social isolation and depression, have been demonstrated repeatedly and authoritatively to have significant negative effects on physical and psychological health and medical outcomes [13, 14]. Most recently, they have been acknowledged as some of the damaging societal consequences of the COVID-19 pandemic [15].

The data presented here are taken from a larger cohort participating in an online UK survey of people living with SLE, which investigated both barriers to employment [16] and the experiences of people in claiming benefits [17]. The findings of these studies may be summarized as follows. First, the nature of lupus is to fluctuate, and its invisibility and associated fatigue create major barriers to maintaining employment, particularly when allied with the unpredictable course of lupus [16]. Second, claiming benefits was experienced as harsh and punitive, causing prolonged and extreme stress, which is known to be a risk factor for relapse in lupus [17]. In both situations (employment and claiming disability benefits), one of the primary difficulties was the lack of recognition of the realities of living with a fluctuating, invisible condition.

When analysing the cohort data, we noted that a significant number of participants (*n* = 72, 18.34%) were currently or previously employed in the NHS. We were interested to explore whether this subgroup were more or less likely to be disadvantaged by the employment difficulties identified in the whole group, because the NHS is a vast organization with significant retraining and redeployment opportunities, which might be expected to demonstrate a higher level of medical literacy than non-health-care organizations and have greater understanding of, and tailor employment interventions to, known illness characteristics. Indeed, the Department of Health report ‘Looking Beyond the Label’ [5] outlines the steps required to employ and retrain more disabled people in the NHS and states that ‘NHS employers’ need ‘to take action to deliver on the Government’s commitments on equality and social inclusion and to demonstrate that they have done so’.

## Methods

After obtaining ethical approval (University of Hull Faculty of Health Sciences Ethics Committee), a cross-sectional online study of UK residents aged 18–75 years, with a self-reported diagnosis of lupus, was carried out to record their experiences in maintaining employment and accessing the UK welfare benefits system. A full description of the methodology and results for the whole cohort are published elsewhere [16, 17]. The survey was posted on the LUPUS UK website using the Bristol Online Software (BOS) and was available from 2 September to 31 October 2017. It was also tweeted by one author (E.W.). One author (S.B.) telephoned all the LUPUS UK regional support group chairs to highlight the survey, and a link to the survey was tweeted by LUPUS UK several times during the data-collection period. It was also highlighted on the blog, and a short article was written for the LUPUS UK national newsletter before the survey was started. This was done to advertise the survey as widely as possible. All responses were anonymous.

The survey consisted of 22 questions. Nine questions were demographic, including one on drug therapy to gauge diagnosis. Fifteen were stem questions, where a quantitative response was required with an opportunity for a free text statement. Ten stem questions related to employment and two to the benefits system.

The topic areas covered were developed with members of the Cambridgeshire Lupus Support Group who live with the illness. A range of demographic data was collected, including education, occupation and employment status. Participants were asked about managers’ and colleagues’ attitudes to their fluctuating illness and to quantify the psychological distress associated with: (a) income loss resulting from lupus; (b) the proportion of income loss from SLE; (c) the degree of fear that participants experienced about sustaining future employment; and (d) experiences of the benefits system. The full questionnaire is published in Supplementary Data S1, available at *Rheumatology Advances in Practice* online.

Thematic analysis [18] of free text comments was conducted independently by all authors, then compared, agreed and summarized. Noting the relatively large number of participants who worked for the NHS, the data for this subgroup were subsequently analysed again separately.

## Results

In the survey of 393 people, 72 (18.34%) were currently (*n* = 51, 12.9%) or previously (*n* = 21, 5.3%) employed by the NHS. The median age range of the sample of people currently or previously employed by the NHS was 45–55 years, the age range of the NHS sample was 18–64 years. One hundred per cent were female.

Table 1 shows sample demographics, including work status, category of work, any reason given for leaving the workforce, current medication, results for the impact of SLE on participants’ mental health, and fears about future earning capacity. Employment was identified very broadly (clinical or non-clinical, community or hospital based), in order to maintain anonymity. The geographical region of individuals was not identified; the sample included people from all areas and countries of the UK. The subgroup included 2 doctors, 32 nurses, 16 administrative staff, 15 allied health professionals or other clinicians, 2 managerial staff, and 5 where the NHS role was unspecified. Three participants were also senior clinical managers. Fifty-eight of the sample described themselves as White British; the balance was dual heritage, Black British, Black Caribbean and other White background. This is lower than the proportion of people with SLE who have Afro-Caribbean or East Asian heritage.

**Table 1:** Demographic characteristics of the sample

Characteristic	Percentage (*n*)
Biological Sex	
Female	100 (72)
Male	0
Age, years	
18–24	4.1 (3)
25–34	11.1 (8)
35–44	27.7 (20)
45–54	43 (31)
55–64	13.8 (10)
Ethnicity	
White British	80.5 (58)
Black British	4.1 (3)
Other White	4.1 (3)
Other	4.1 (3)
Dual heritage	2.7 (2)
Asian British	1.3 (1)
Black Caribbean	1.3 (1)
Asian	1.3 (1)
Time since diagnosis, years	
<1	2.7 (2)
1–5	30.5 (22)
6–10	22.2 (16)
11–15	13.8 (10)
15+	30.5 (22)
SLE drug treatments	
None	2.7 (2)
1	16.6 (12)
2	29.1 (21)
≥3	45.8 (33)
Did not state	5.5 (4)

Sixty-five participants (91.5%) were taking at least one drug specific for SLE treatment. Fifty-three (74.6%) people were on two or more disease-specific drugs for SLE, including prednisolone or another immunosuppressant. Fifty-five (77.5%) of the cohort were on HCQ; two people were on no medication, and four did not answer the question. This suggests that this cohort predominantly had moderate to severe SLE.

The main themes identified for this cohort and employment in the NHS were as follows:

1. The direct negative effect of SLE on employment (*n* =22).
2. Lack of employment support from the NHS (*n* =20).
3. Changes in employment that supported better health (*n* =7).
4. Examples of good NHS practice supporting continued employment (*n* =4).
5. Fears about future ability to work and financial security (*n* =5).
6. Feelings of shame, guilt and being a burden (*n* =4) to family and colleagues.

The major themes are discussed individually below, and data are presented in Supplementary Data S2, available at *Rheumatology Advances in Practice* online. It is clear that several of the identified themes are relevant to many participants.

The characteristics of lupus that make employment difficult to maintain, identified by Booth *et al.* [16] (fluctuation, invisibility and fatigue), were also representative of this subgroup and integral with data presented for each individual and are not discussed or presented separately. The difficulties with the benefits system published by Price *et al.* [17] are also representative of the subgroup (i.e. the process of claiming benefits was characterized as punitive, demeaning and stressful, with a lack of understanding of the fluctuating nature of lupus, often exacerbating the severity of the illness).

### Theme 1: direct negative effects of lupus on employment (22 participants)

Our data are consistent with other evidence showing that SLE has a directly detrimental effect on employment opportunities of participants (see Boxes 1a, b, c, d in Supplementary Data available at *Rhematology Advances in Practice* online). Twenty-one members (29.16%) of this sample of skilled people of working age had left employment of any sort: eight had been dismissed on capability grounds, six had taken medical retirement, one had left voluntarily, one had been made redundant, two were planning to leave imminently, one described herself as ‘medically resigned’, and one had left without describing the circumstances. Leaving work had caused all these women significant emotional and financial distress.

Participant 371: Devastated and is still difficult to accept…

Participant 095: I’m out of a well-paid job to go onto a very small pension.

Twenty (27.8%) participants had moved to part-time work for health reasons, rather than individual choice, which caused financial concerns from an unplanned and unwelcome reduction in income.

Participant 171: All in all, I lose about £1000 per month as a result. I am a single woman with a mortgage and bills, so you can imagine the impact this has had on my life.

Many of these participants were young, with many years of potential working life left.

Participant 15: …becoming significantly disabled at thirty is hard, giving up work has been essential but a disaster at the same time, if that makes sense…

There were no participants who experienced a financial improvement or promotion in their careers. As a result of having lupus, many worked at a lower seniority than commensurate with their skills and training and undertook fewer hours than they would have chosen. Only one person (participant 164; Box 3, Supplementary Data S2, available at *Rheumatology Advances in Practice* online) felt that she had changed her work–life balance for the better because of lupus.

Participant 164: Love my current job and life!… Probably would still be in full-time job I disliked if it weren't for lupus.

This change was accompanied by a significant financial loss that many people would not be able to sustain. Grief, anger and distress were the most common reactions.

Participant 380: I was very angry. I worked hard to achieve my career aims … now I feel very fortunate just to be able to work at all. My retiring salary was 68 K. I now earn with pensions and work around 35 K.

The financial losses compounded feelings of guilt at not making a contribution to family and household.

Participant 29: I now earn the minimum wage of £7.50 an hour when I used to earn £13 an hour, now work 4 h a week when I used to work 12, sad. I used to work 40 h a week and sleep in two nights. I had to give up the job I loved. I have to rely on my husband giving me money each month.

The lack of structure and sense of purpose unemployment can bring was repeatedly mentioned by participants.

Participant 283: I do miss being able to work and have a sense of purpose.

Participant 331: The loss of my career, friends, living with a chronic disease which changes with the wind, creating new and distressing symptoms, the loss of my home and lifestyle all combined to cause my mental health to suffer….

In summary, lupus caused individuals significant career damage, leading to reduced incomes (sometimes poverty), diversion from their chosen career path, accepting demotion below the seniority or complexity of work that their education, competence and training would warrant, and living with a constant background of anxiety about the possibility of not being able to maintain any work in the longer term or working in a way that damaged their health further.

### Theme 2: lack of employment support from NHS (20 participants)

The examples of good practice were sadly outnumbered by participants who felt that the behaviour of NHS employers, managers and colleagues and the support offered by their organization had been less than helpful or effective at retaining them in employment.

Their reasons (detailed in Boxes 2a and 2b in Supplementary Data S2, available at *Rheumatology Advances in Practice* online) included a lack of knowledge about SLE, a deficit of compassion and understanding of the condition from managers and colleagues, allied with an inflexibility about working hours and patterns from managers. Generally, there was a lack of awareness, insight and understanding of the fluctuating nature of SLE.

As one experienced clinician (participant 093) reported:

My employer did not even consider reasonable changes to my working hours during a flare. I have repeatedly asked for adjustments to prevent this happening, accepting … income drop, but my employer will not consider this … and feels I am unreasonable to request such changes.

She added:

I have found the mental stress of dealing with my employer very damaging.

The all-or-none view of health prevented this clinician contributing what she could during periods of impaired health; she was forced to be entirely absent from work (leading to a poor sickness record) rather than her preferred approach, which was to compromise by reducing her income and working hours temporarily, maintaining her work record and some feeling of contribution, and retaining skills within the NHS.

This lack of flexibility, at times, verged on an explicitly punitive approach.

Participant 093: My employer has stated openly that my lupus is an excuse for me to pick and choose what shifts I want.

Participant 044: …too much time off work resulted in threats from my employer (NHS) of loss of pension rights and dismissal….

Twenty-two people worked part time because of SLE. It was common for them to forego the best-paid part of their employment (out-of-hours working) to manage their health. This need to reduce hours was sometimes presented as a threat.

Participant 171: I was full time and doing on call before I became ill. I also had the opportunity of overtime on weekends. As a result of my sickness record, I was forced into reducing my hours. I was told if I didn't I could risk losing my job.… Devastated. There has been a huge financial impact on me, but also it was damaging from a psychological point of view….

### Theme 3: successful changes in employment (seven participants)

**Fig. 1:**
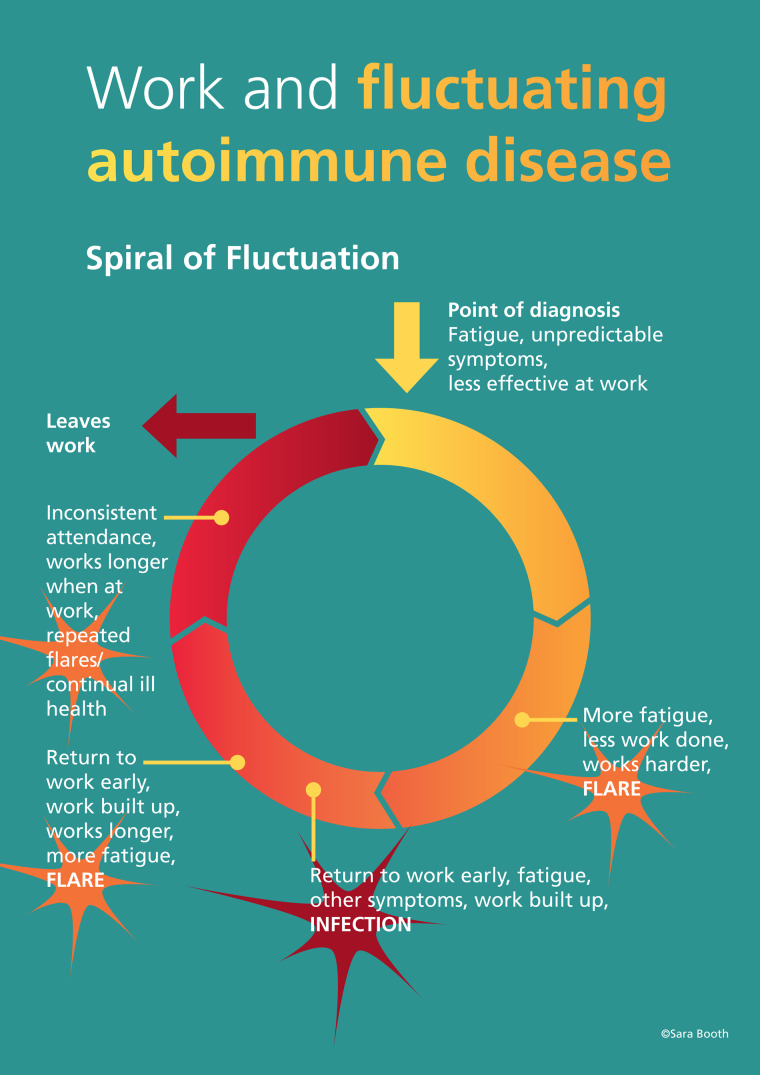
Work and fluctuating autoimmune disease

There were examples of people who had made a successful change into another type of employment, either within the NHS (four participants) or outside (three participants; Box 3, Supplementary Data S2, available at *Rheumatology Advances in Practice* online). The changes that improved health and quality of life included: (a) a reduction in hours worked, even if working full time (e.g. unpaid, unpredictable overtime stopped); (b) changes in working pattern to allow flexibility and workload predictability; (c) self-employment with the option of home working; or (d) working fewer hours at one session. They had actively avoided the exhaustion–flare–early return to work–relapse cycle so commonly described by others (Fig. 1). All participants commented on the positive value of maintaining gainful and productive employment.

Three participants in this group reported significant drops in income that would be unsustainable for many people (participants 123, 164 and 356), particularly if a sole breadwinner. As participant 123 remarked, ‘Had I remained single with a mortgage, I would have been very worried about my finances and lifestyle…’.

Participant 123 noted again the lack of knowledge of lupus within her hospital Trust:

I work in health, and although previous employers knew, I don't think they understood. They just let me get on with my work and as long as there were no complaints left me alone. Still did exactly the same work as someone without lupus. No adjustments made.

### Theme 4: good practice within the NHS, which helped participants to stay employed within the NHS at the time of the study (four participants)

The data show that supportive, proactive management can help people to remain in work (Box 4, Supplementary Data S2, available at *Rheumatology Advances in Practice* online). The examples of good practice that enabled people to stay employed within the NHS in their preferred role were as follows: (a) a proactive, affirming, supportive attitude towards the individual; (b) demonstrations from managers and colleagues that they understood the nature of SLE; (c) managers enabling individuals to change their workload and pattern according to their condition (e.g. reducing shift lengths, home working, changing shift patterns; and (d) implementation of interventions from occupational health services (OHS).

Participant 296: I was reviewed by Occupational Health after I became unwell, and the adjustments to my work have made an enormous difference. Having a day off mid-week means I only work 2 days in a row and helps me manage the fatigue. I am fortunate that my job is well paid and I can afford to work reduced hours (in postgraduate training post).

OHS interventions were rarely mentioned otherwise in this NHS cohort.

Again, participant 296 had had to take a cut in salary, which will affect her long-term pension, which will be much smaller again if she is required to retire early. Many people would find it difficult to take an unplanned 20% cut in salary.

Even where participants described a mostly supportive reaction from NHS employers, they often felt there was room for improvement in the system itself.

Participant 72: ‘…(managers) to a certain extent are supportive, but I still feel much more could be done…’

Participant 215: But some of my co-workers don't understand. Some new co-workers think I am just lazy, which is very frustrating….

Participant 296: My employer uses the Bradford factor, which discriminates against people with chronic illness who need to take multiple short periods of sick leave….

The Bradford factor is a calculation used by human resources departments to assess the impact of repeated short-term absences from work; 2 weeks off in one period is considered less disruptive than 10 working days taken as several discrete episodes. The Bradford factor is calculated as the number of unrelated absence periods squared, multiplied by the number of days absent. For example, 10 days of absence in the reference period (e.g. 1 year) could occur as:

- One absence of 10 days, which would have a Bradford factor of [(1 × 1) × 10] = 10.
- Five absences of 2 days, each which would have a Bradford factor of [(5 × 5) × 10] = 250.
- Ten absences of 1 day, each which would have a Bradford factor of [(10 × 10) × 10] = 1000.

It has been characterized by UNISON (a health Trades Union) as a ‘blunt instrument that takes little account of what is happening to an individual’s health’ [19].

Although most participants in this group reporting good human resources practice in the NHS were in work and mostly felt supported, there remained significant anxieties, both about working harder than was good for their health for financial reasons and about their capacity to be able to work until the current retirement age of 67 years.

### Theme 5: fears about a future ability to work and income

Even those in work had substantial fears about being able to continue in work until retirement age (Box 5, Supplementary Data S2, available at *Rheumatology Advances in Practice* online).

Participant 110: As I get older, I worry I may get more complications as a result of lupus and the medications I take to treat it. This could make working full time difficult.

Participant 137: …would like to cut hours but worry about what might happen in the future if I have periods of absence due to my lupus flares, so working full time just now while I can manage.

Participant 163: I have a very small Health Service Pension and so I worry about my future finances. I am fortunate to have a husband with a modest pension, but I still feel vulnerable financially as I get older.

Participant 185: I am constantly worrying about the impact of the stress of the job on my body, as historically stress has caused me to have quite severe lupus flares and hospitalized with sepsis on more than one occasion. I worry that if I am off sick too much due to my lupus, employers will dismiss me….

There was no suggestion that any of these concerns were discussed with their employers or OHS with the idea of anticipating these issues and planning for them in the longer term, although the SLE literature suggests increasing morbidity with time in those on long-term medication or with continuing disease activity [7, 8]; therefore, it could be anticipated and planned for.

### Theme 6: feelings of shame, guilt and of being a burden (four participants)

Many participants expressed feelings of shame at their illness and the limitations it imposed; of being a burden to their family and colleagues (Box 6, Supplementary Data S2, available at *Rheumatology Advances in Practice* online). Participants reported making significant efforts to make up for this and the accompanying guilt at being unable to perform their work and home roles as well as they would like. This, in turn, led to greater exhaustion, resulting in a vicious circle of further ill health.

As participant 118 said: …Determination to try and do the best I can under the circumstances … to be as productive as colleagues. Due to this I have been pushed to the limit mentally and physically and had to resign as feel not firing on all cylinders and … a failure….

## Discussion

Despite the recruitment and retention problems in the NHS, its long-term stated aim of employing more people living with LTC/Ds and a commitment to flexible working patterns [4–6], our data demonstrate that people living with SLE and working in the NHS are not, in the main, enabled to remain productive members of the NHS or any other workforce. This premature ending of individuals’ careers, with loss of their skills to the NHS and wider society, and with others working in a less than optimal ways, has damaging consequences for the wider economy, the NHS and, more destructively, for NHS employees with lupus and those closest to them. It also increases societal costs (with the need for greater use of inadequate benefits) without relieving stress or poverty and leads to the health problems associated with long-term unemployment [14].

Our data highlight, again, the clearly and repeatedly documented fact that lupus has profound negative consequences on the life chances of people who live with it; people in their peak years for education and employment. Many participants worked fewer hours than they would choose and/or worked at a level below the seniority (and pay) their educational attainments and experience warranted. Many without professional qualifications, working in areas with high physical demands, obtained no help to transition to other employment. Most of the financial and psychological burdens were carried by the individual, largely hidden and unacknowledged by the employer, unions and wider society.

It is inconsistent with an evidence-based approach that although the problems of disability at work, premature retirement and unemployment in SLE are well documented there has been no concerted effort to address them. One Department of Health report, now >20 years old, addresses fluctuating illness in a short section [5] by noting, ‘Many employees with long term medical conditions will be able to continue to work provided they have regular work patterns and are able to take breaks. Flexibility over timekeeping and working hours may also be required… Some progressive or fluctuating conditions may result in variations to a person’s stamina over the day. Flexibility over working hours can often hold the answer’.

The same report also recommends that employers recognize that people with LTC/Ds are not a homogeneous group. This is important because disability support is often seen as simply a case of providing equipment or changing the workplace environment; the more complex issues of changing work patterns or redeploying or retraining individuals when they are simply unable to continue reliably in their current role are less commonly addressed and are certainly under-researched [18, 20]. One participant (participant 123) referred to another solution mentioned in the Department of Health report [4], ‘time banking’ to ‘allow individuals to bank overtime to compensate for future absences’.

In this study, many who left the NHS did not work elsewhere. Many tried to claim benefits (unsuccessfully) and lived in poverty or relied on other members of their families for support, feeling guilty and a burden. Others were working more intensely or in a pattern that would mitigate against them maintaining optimal health in the long term but saw no other choice if they were to earn enough to live on.

The NHS needs clinicians to provide a consistent level of performance and attendance to give patients the best treatment and for all employees to have equitable levels of work. A proactive approach is therefore needed to help people with a fluctuating condition maintain satisfactory work. If one type is no longer suitable, retraining or redeployment might be necessary. The present study demonstrates that employees with SLE (in the NHS) are not generally given the informed support and adjustments they need to achieve this and that there is little research evidence available for doing this. This must change if the NHS is to achieve a truly diverse workforce, protect the health of its employees and, not least, retain valuable skills and experience. This might require individuals to have an individualized job plan, outside normal parameters [20, 21], or be redeployed or retrained to attain this. With flexibility being a right for all members of the NHS workforce, under the new plans there should be no difficulty in this being given to an individual with an illness, but where an unusual individualized job plan is devised, communication to all team members will be important because, as our data show, some managers, colleagues and human resources departments believe that invisible illness can be a method used by manipulative individuals to achieve better working patterns than are routinely available.

It is interesting to note, at this point, the emerging data, personal stories and discussions about the fluctuating fatigue and brain fog and other symptoms that accompany long COVID (which, at least in part, is a post-inflammatory autoimmune condition). These discussions have highlighted the reality of these symptoms in the context of conditions such as SLE [22], but our participants demonstrate that symptoms such as these are often disbelieved, minimized or simply dismissed by managers and sometimes colleagues [23].

The data suggest that this lack of support for people with fluctuating, invisible disease is related to factors both within and outside NHS organizational control and/or that of individual NHS managers. The factors are outlined in Table 2.

**Table 2:** National Health Service and other factors inhibiting employment of people with SLE

Possible NHS-related factors	Factors outside the control of the NHS as an employer
Ignorance of managers about SLE and its known impact on employment, which might also to apply to other fluctuating conditions	A lack of vocational rehabilitation for people with SLE, with a paucity of research evidence to guide effective work interventions [23]
A reactive, at times punitive, approach to employees in difficulty, including those with previously consistent and unblemished work records	The high prevalence of fatigue in SLE and the lack of effective management strategies to palliate the symptom after maximal disease-focused treatment [20]
An apparently low use of OHS or, where used, a tendency to overlook OHS recommendations and, possibly, a lack of informed OHS support	A focus in equality reports/legislation on the provision of equipment or alterations in the physical work environment rather than examination of work patterns [4, 18]
An apparent lack of redeployment and retraining opportunities or knowledge of these	The lack of data (and understanding) on ways to mitigate the impact of fluctuating, invisible illnesses in the workplace [15, 18, 20, 23]
Scepticism about the symptom of fatigue and its impact on work disability	
The demeaning nature of the attitudes of some colleagues to invisible illness associated with fatigue	
NHS ill-health retirement pension arrangements that penalize intermittent work within the NHS and related organizations [21] for those with the most severe illness	
Inflexible sick-leave arrangements, which, although generous in length compared with most organizations, are predicated on an all-or-none pattern of health [22]	

NHS: National Health Service; OHS: occupational health services.

Possible NHS-related factors that reduce the chances of people with SLE continuing to work within the organization include poor human resources and people management knowledge, skills and attitudes, which could be overcome within organizations with suitable training and policy changes. This could also have implications for the continued employment of those with long COVID [24].

Factors outside the control of the NHS as an employer are often related to the lack of societal resources available to enable people to manage the psychosocial impacts of chronic non-malignant disease. These societal deficits perpetuate an inaccurate binary model of health, which mitigates against employment models being explored that, for example, would allow people with LTC/Ds to receive income from part-time, fluctuating work supplemented by state benefits or work-based pensions. The Tier 2 regulations for the NHS pension are punitive to re-employment in the NHS or related organizations [25, 26]. There is a lack of a proactive approach to keeping people in work, including retraining or redeployment into suitable, equivalent employment if current work is damaging health or if individuals are unable to maintain consistent levels of work. There is also a need for specialist OHS, rehabilitation and vocational rehabilitation services to support people with fluctuating, complex and particularly rare illnesses.

Recognizing the pattern of impairment for an individual is crucial to fit the intervention to the work disability being addressed and the individual needs of the person with the disability. From the data on this SLE cohort, and the clear evidence in the literature that fluctuation, invisibility and fatigue have a detrimental impact on employment [16], we propose that characterizing these barriers in a descriptive term for the conditions, such as fluctuating, invisible conditions with constitutional symptoms (FICCS), might make it easier for managers and OHS and vocational rehabilitation services to recognize these attributes as needing to be addressed actively in employment, and for occupational health research to address these issues in diseases with similar characteristics, such as multiple sclerosis, long COVID, IBD and other autoimmune conditions. Each characteristic needs solutions to help people stay in work and improve other areas of their lives.

Our data suggest that the changes in work practices that seemed to make a positive difference to people with lupus maintaining employment included the following: (a) a positive problem-solving approach from managers; (b) reducing hours where requested; (c) changing the work content for individuals, making it less physically demanding (e.g. reducing the proportion of clinical work and introducing non-clinical elements); (d) reducing shift lengths; (e) changing work patterns; and (f) enabling flexibility in the place of work (e.g. home working). People with lupus seem to benefit from a predictable, contained workload that can be worked flexibly. Some may need to retire and claim benefits when they need to work very part time or irregularly. Sadly, the process of claiming benefits is damaging for this NHS group, as described by Price *et al.* [17]. Those in this study who could retire and return to the NHS intermittently benefitted psychologically, although they were still significantly worse off financially. NHS ill-health retirement regulations were changed in 2008 such that those who retire with the most severe illnesses are penalized if they worked intermittently for the NHS and a wide range of other related employers [25, 26], preventing them from using their skills and supplementing their income.

In this cohort, there were few mentions of OHS, although these are promised to be extended in the latest NHS People Plan. It is clear that many promises and recommendations made by NHS employers and the Department of Health in older reports have not been adhered to [27, 28]. There is no recognition of previous attempts at introducing flexible working and improving OHS in current reports nor is it clear how it will be ensured that these new recommendations are implemented. It would be expected that improved OHS (with vocational rehabilitation) and greater opportunities to work flexibly could play a significant role in retaining people with complex health needs, particularly if used early in the disease course. Where OHS recommendations were followed for this cohort, they were reported as helpful. Sometimes recommendations were ignored; even simple reductions in hours worked. Participants often mentioned being ‘threatened’ if their work performance did not improve, sometimes when occupational health recommendations had also been disregarded. Given that SLE is a rare illness and follows a fluctuating course, some OHS might not be able to advise appropriately; one solution might be the provision of regional OHS of multidisciplinary rehabilitative teams to ensure that people with rare illnesses are not penalized by lack of knowledge of their condition by local services.

It is also clear that inadequate symptom control allied with employment difficulties causes psychological and financial distress to people with SLE. A psychosocial support infrastructure is not available to people with non-malignant disease (it is an expectation in cancer medicine, although not always provided). It is unclear why there is such a discrepancy in provision of these important treatment strategies in people with life-long, highly symptomatic illnesses that have such a profound effect on life chances.

Steadman *et al.* [20], in a rare review of the occupational aspects of fluctuating conditions, stated that ‘static and inflexible company policies can form a barrier to making changes that would benefit the employee and ultimately the employer … any organization that wishes to take a proactive approach to supporting employees with fluctuating health conditions must allow space to both employees and their manager, to allow changes to be made that support the needs of both parties—allowing productive work to continue while meeting the broader organizational needs’. Steadman *et al.* [20] made a number of recommendations, including an ‘employee passport’ (currently being introduced in medical schools and some areas of the NHS, but not yet evaluated for effectiveness) and stated that ‘organisational culture and stigma provide a barrier to work for those with fluctuating health conditions, marked by a poor understanding by employers and colleagues about fluctuating conditions’. This is consistent with our findings in the present NHS cohort. Interestingly, many of the issues highlighted in our study, including the need for individuals to conceal their illness, the experience of being harassed and demeaned by both managers and colleagues, and the lack of appropriate support, have been reported again in a recent survey on disabled doctors carried out by the British Medical Association [29]. Fluctuating, invisible conditions were stated to be particularly problematic.

As one of our participants (participant 293), who worked in human resources, said:

Concerned that I might not be able to support my family if I get ill. I have not told my current employer as it is still career limiting, I know—I'm in HR [human resources]. Do not discuss (with colleagues).

Our data suggest that employees with SLE (and other FICCS) need to engage with a system that is potentially causing them physical and emotional harm without a focused attempt to understand the rehabilitation and employment support needs of this group. We propose that the clinical effectiveness and cost-effectiveness of different sorts of early vocational rehabilitation and specialist occupational health support for people with lupus (and other diseases with profound effects on employment) should be researched more thoroughly. The impact on employees doing different sorts of work (e.g. ward nurses with a high level of physical work over a long shift) also needs careful examination to inform redeployment and retraining in the service. We are about to examine what can be provided currently in the NHS within a research study. Mixed-methods longitudinal studies of those with moderate to serious SLE returning to work would be helpful. Some way of following the progress of those with SLE (and other FICCS) over years would be key to the retention of people in the workforce over the life-time course of LTC/Ds with accumulating morbidity.

Current data [20, 21, 27] suggest that effective workplace rehabilitation and adjustment might prevent people leaving employment, potentially improving the health of people with diseases such as SLE whilst reducing health and social costs for society. As our data show, young people (e.g. participant 240) are concerned about never being able to work because of concerns about their health and gaps in their curriculum vitae. These issues are not being addressed proactively, potentially losing >40 years of working life and income in someone 18 years of age. Unions and other representative bodies (e.g. the Royal Colleges) need to take a more active interest in the needs of disabled members, examining carefully the impact of proposed changes in policy and regulation on vulnerable groups (e.g. the 2008 pension changes) [25, 26].

The COVID-19 pandemic has demonstrated that there are many different work patterns that enable people to carry out clinical and other NHS work safely. NHS employees living with immunosuppressive illness have been moved to remote consultation duties, and home working has become widely accepted. Retired clinicians have returned to the workforce, and both currently employed and other clinicians have been moved temporarily to duties outside their usual specialty. This demonstrates that these changes in working content and pattern are possible and, outside an emergency, where they could be done in a more considered way with greater time for training, could offer ways to redeploy, rather than fire or retire, NHS staff. The NHS could become a national leader in this area for medical conditions where unemployment is known to be high [27, 28]. The detrimental impact of unemployment, the need for active rehabilitation of those with post-COVID inflammatory fatigue and brain fog, and the possibilities for flexible furloughs from work have been part of the current national discussion and are particularly relevant to this cohort. Long COVID rehabilitation clinics have been established rapidly, whereas they do not exist for people with SLE or for many people with other long-term conditions or for those requiring rehabilitation after accidents or other surgical treatments [22, 30], which is inequitable.

The limitations of the present study include the fact that it is not representative of the population of people with lupus who work in the NHS. There is an important group of people who work for the NHS but who are not employed by the NHS; they were lacking from our sample. These are people such as cleaners and catering staff, who are employed by companies that are contracted to provide these services to the NHS. People employed in insecure, low-paid work are least likely to be able to negotiate their terms and conditions of employment. Future studies need purposively to sample those in manual, low-paid, insecure and zero-hours occupations.

Additionally, although no exact numbers exist for the proportion of people of different ethnic groups employed in the NHS, the number of research participants with Afro-Caribbean heritage or East Asian heritage (none specified) were below what we would expect in a cohort of people with SLE. There were more people with moderate to severe illness requiring immunosuppression in this cohort who would be anticipated to need employment support. The sample was of volunteers responding to a survey, not a purposive sample representative of the wider population of people with lupus. People with more severe disease would be expected to have more employment difficulties and possibly greater motivation to answer the survey. Further specific research on overcoming the employment difficulties of people with SLE (and other FICCS) is needed. It needs to include purposive sampling to recruit a representative sample of individuals in different work settings (including self-employment) that are characterized carefully in order to define the sorts of employment practice that help to sustain employment in people with SLE. Longitudinal studies reflecting the changes in work that might be needed with the accumulating morbidity of medication and disease in those with SLE are also needed.

The NHS cohort did not include enough medical staff at different stages of their career (e.g. medical students and consultants). It seems easier for those in training grades [29] to obtain changes to their job plan with personal circumstances. Otherwise, a wide range of those working in clinical and non-clinical roles responded. There were no men, and given that they have more severe disease they are an important omission, which purposive sampling could overcome.

The strength of the sample was that it included people from across the age ranges. Although most were >35 years of age, this would be expected in a chronic, lifelong illness that has peak onset in youth. Respondents came from all over the UK. NHS employees would be used to using computers in every part of their work; therefore, there was no concern about excluding those who were unused to computers.

It is time to move on from documenting the negative impact of SLE on educational and employment opportunities for those who live with it, to find solutions that will benefit not only individuals and their families but also health services and wider society and, it is likely, others with FICCS [30–32]. The aspirations of successive NHS reports, published over many years, need to be implemented rather than endlessly publicized. The most recent NHS initiative addressing disability is the setting of workforce disability equality standards (https://www.england.nhs.uk/about/equality/equality-hub/wdes/), requiring information to be sent centrally from UK NHS organizations but with no change of practice required and apparently without recognition of the diversity of needs of people with different sorts of disabilities. The detailed work on finding solutions to employment barriers for people with individual diseases or disease groups has not been carried out.

The punitive approach to managing those with debilitating long-term conditions needs to be eradicated by better training of and support for managers and the use of excellent, specialist OHS and vocational rehabilitation services. The NHS needs to support rehabilitation for people with all sorts of illnesses and accidents in the same way that it has rapidly provided rehabilitation for those with long COVID [22, 30]. This study suggests that the if NHS continues to use its current workforce practices, it will not retain those of its staff with persisting symptoms from long COVID, which has many similarities with SLE in its reported impact on functioning. The participants stressed the need for greater awareness of lupus and its impact on employment. This could be included in position statements of academic lupus organizations and charities [30], and the medical faculties of vocational rehabilitation and occupational health need to highlight, for example, counter-productive employment practices, such as the Bradford factor and the ill-health retirement regulations [19, 25]. High-quality research is needed to test early employment support [20, 27, 31, 32] that will overcome the current barriers that people with SLE and other FICCS face in trying to live as healthy, productive and normal lives as possible, which will benefit not only themselves but also the NHS and wider society.

## Supplementary Material

rkab019_Supplementary_Data

## References

[1] NHS.gov.uk NHS Constitution for England 2015 gov.uk. https://assets.publishing.service.gov.uk/government/uploads/system/uploads/attachment_data/file/480482/NHS_Constitution_WEB.pdf (7 June 2020, date last accessed).

[2] NHS.gov.uk NHS Workforce Statistics. https://digital.nhs.uk/data-and-information/publications/statistical/nhs-workforce-statistics/july-2020 (22 October 2020, date last accessed).

[3] Interim NHS People Plan 2019. https://www.longtermplan.nhs.uk/wp-content/uploads/2019/05/Interim-NHS-People-Plan_June2019.pdf

[4] NHS. We are the NHS: People Plan for 2020–2021 – action for us all. July 2020. https://www.england.nhs.uk/publication/we-are-the-nhs-people-plan-for-2020-21-action-for-us-all/ (28 October 2020, date last accessed).

[5] Department of Health. Looking Beyond Labels; Widening the Employment Opportunities for Disabled People in the New NHS Department of Health 2000.

[6] Department of Health. Improving working lives in the NHS (IWL). London: Department of Health, 2001.

[7] D'Cruz DP , Khamashta MA, Hughes GR. Systemic lupus erythematosus. Lancet 2007;369:587–96. 17307106 10.1016/S0140-6736(07)60279-7

[8] Durcan L , O’Dwyer T, Petri M. Management strategies and future directions for systemic lupus erythematosus in adults. Lancet 2019;392:2232–43. 10.1016/S0140-6736(19)30237-5 31180030

[9] Yelin E , Cisternas MG, Pasta DJ et al Medical care expenditures and earnings losses of persons with arthritis and other rheumatic conditions in the United States in 1997: total and incremental estimates. Arthritis Rheum 2004;50:2317–26. [PubMed: 15248233] 15248233 10.1002/art.20298

[10] Turchetti G , Yazdany J, Palla I, Yelin E, Mosca M. Systemic lupus erythematosus and the economic perspective: a systematic literature review and points to consider. Clin Exp Rheumatol 2012;30(4 Suppl 73):S116–22. PMC3714226 23072767

[11] Drenkard C , Bao G, Dennis G et al Burden of systemic lupus erythematosus on employment and work productivity: data from a large cohort in the southeastern United States. Arthritis Care Res 2014;66:878–87. 10.1002/acr.22245 24339382

[12] Bultink IEM , Turkstra F, Dijkmans BAC, Voskuyl AE. High prevalence of unemployment in patients with systemic lupus erythematosus: association with organ damage and health-related quality of life. J Rheumatol 2008;35:1053–7. 18381792

[13] Baker K , Pope J, Fortin P et al; 1000 Faces of Lupus Investigators and CaNIOS (Canadian Network for Improved Outcomes in SLE). Work disability in systemic lupus erythematosus is prevalent and associated with socio-demographic and disease related factors. Lupus 2009;18:1281–8. 19854811 10.1177/0961203309345784

[14] Marmot M. Health equity in England: The Marmot Review 10 years on. BMJ 2020;368:m693. 32094110 10.1136/bmj.m693

[15] Douglas M , Katikireddi SV, Taulbut M, McKee M, McCartney G. Mitigating the wider health effects of covid-19 pandemic response. BMJ 2020;369:m1557. 32341002 10.1136/bmj.m1557 PMC7184317

[16] Booth S , Price E, Walker E. Fluctuation, invisibility, fatigue – the barriers to maintaining employment with systemic lupus erythematosus: results of an online survey. Lupus 2018;27:2284–91. 30451638 10.1177/0961203318808593 PMC6247450

[17] Price E , Walker E, Booth S. Feeling the benefit? Fluctuating illness and the world of welfare. Disabil Soc 2020;35:1315–36.

[18] Braun V , Clarke V. Using thematic analysis in psychology. Qual Res Psychol 2006;3:77–101.

[19] https://www.unison.org.uk/content/uploads/2014/09/TowebFact-Sheet-on-the-Bradford-Factor2.pdf.

[20] Steadman K , Shreeve V, Bevan S. Fluctuating conditions, fluctuating support: Improving organisational resilience to fluctuating conditions in the workforce. The Work Foundation (Lancaster University), 2015.

[21] Bingham C , Clarke L, Michielsens E, Van de Meer M. Towards a social model approach? British and Dutch disability policies in the health sector compared. Personnel Rev 2013;42:613–37.

[22] Wade DT. Rehabilitation after COVID-19: an evidence-based approach. Clin Med 2020;20:359–64. 10.7861/clinmed.2020-0353 PMC7385804 32518105

[23] The jobs that covid crushed. https://www.bma.org.uk/news-and-opinion/the-jobs-that-covid-crushed (19 January 2021, date last accessed).

[24] The BMA – doctors with long covid. https://www.bma.org.uk/news-and-opinion/doctors-with-long-covid) (20 January 2021, date last accessed).

[25] Tier 2 pensions regulations. https://www.nhsbsa.nhs.uk/nhs-pension-scheme-regulations (12 October 2020, date last accessed).

[26] Sickness absence regulations. https://www.nhsemployers.org/tchandbook/part-3-terms-and-conditions-of-service/section-14-sickness-absence-england (19 October 2020, date last accessed).

[27] Bisung E , Elliott SJ, Clarke AE. Non-pharmacological interventions for enhancing the working life of patients with lupus: a systematic review. Lupus 2018;27:1755–6. 10.1177/0961203318777119 29768969

[28] Skinner D , Saunders MNK, Duckett H. Policies, promises and trust: improving working lives in the National Health Service. Int J Publ Serv Manag 2004;17:558–70.

[29] BMA London. BMA Equality, Diversity and Inclusion Group. Disability in the Medical Profession, Survey Findings 2020. https://www.bma.org.uk/media/2923/bma-disability-in-the-medical-profession.pdf

[30] Booth S. Improving medical outcomes in lupus: enhancing the effectiveness of the medical interview and improving patient support. Rheumatol Adv Pract 2020;4:rkaa066. 33376946 10.1093/rap/rkaa066 PMC7750716

[31] Scofield L , Reinlib L, Alarcón GS, Cooper GS. Employment and disability issues in systemic lupus erythematosus. Arthritis Care Res 2008;59:10. 10.1002/art.24113 18821664

[32] Agarwal N , Yasui NY, Kumar V. Lupus: vocational aspects and the best rehabilitation practices. J Voc Rehabil 2015;43:83–90.

