# Supplementary material for: ‘When I asked for help and support it was not there’: current NHS employment practice and its impact on people with systemic lupus erythematosus
Source: Rheumatol Adv Pract. 2021 Mar 12;5(1):rkab019. doi: 10.1093/rap/rkab019 (PMC8058394; doi:10.1093/rap/rkab019)
Supplement: rkab019_Supplementary_Data [file rkab019_supplementary_data.zip › 20-142 Supplementary Data S1.pdf]

UNIVERSITY OF HULL

Employment and Lupus

Page 1: Page 1

Employment and Lupus

We all know that lupus is very difficult to live with and affects all areas of our lives. One of the most difficult issues for people living with a relapsing and remitting condition like lupus is employment. Lupus comes and goes in an unpredictable way and this makes maintaining a regular job very difficult. If you are very young when lupus affects you, it could prevent you beginning your employment journey.

This research project, based at the University of Hull, explores your experiences of living and working with lupus. We hope the work will inform employment policy and practice.

The questionnaire should take between 15-30 minutes to complete, but please feel free to only answer the questions that you feel able/comfortable to.

Once you have started the questionnaire you are, of course, free to withdraw/stop at any time (your responses/answers will not be recorded until you click ‘finish’. After this point, you will be unable to retract your questionnaire).

Your responses are anonymous (there is no way to identify you from your responses), but please avoid using any personal identifying information in your answers to the questions.

If you find completing the questionnaire distressing and would like some support, please do contact our research partners, LupusUK, who can direct you to a range of support services. They can be found here http://www.lupusuk.org.uk/

The data collected for the study will be used in academic publication and conference proceedings and may help inform policy on employment for people with lupus. This may

include quoting your responses.

If you would like more information about the project, please contact us at the email addresses below.

If you are happy to continue and have read and understood the information above, please tick the box below.

Thanks again for taking the time to help us with this work.

Dr Sara Booth

Professor Liz Walker e. walker @hull.ac.uk

Dr Liz Price e.price@hull.ac.uk

1. Please indicate that you have read and understood the information above

- [ ] I have read and understood the information above

Page 2: About You

2. What is your age?

- ◯ 18 - 24
- ◯ 25 - 34
- ◯ 35 - 44
- ◯ 45 - 54
- ◯ 55 - 64
- ◯ 65 - 74
- ◯ 75+

3. How would you describe your gender?

- ◯ Female
- ◯ Male
- ◯ Agender
- ◯ Other

4. Your Education. Please indicate below the highest level of qualification you hold.

- ☐ GCSEs / O-levels
- ☐ A Levels
- ☐ Higher National Diploma
- ☐ Undergraduate degree or equivalent
- ☐ Post-graduate degree
- ☐ Other

5. Which of the following best describes your ethnic group?

- Black British
- Black African
- Black Caribbean
- Other Black background
- White British
- Other White background
- Asian
- Asian British
- Chinese
- Arabic
- Dual heritage
- Other ethnic background

6. Which area of the UK do you live in?

- South East England
- South West England
- North East England
- North West England
- The Midlands
- London
- East of England
- Scotland
- Wales
- Northern Ireland

7. How long have you had lupus?

- Less than 1 year
- 1 - 5 years
- 6 - 10 years
- 11 - 15 years
- More than 15 years

8. Do you take medication for your lupus?

- Yes
- No

8.a. If you answered 'yes', please could you tell us what medication you take.

Page 3: Your Working Life

9. Are you in paid employment?

- ◯ Yes
- ◯ No

9.a. Are you self-employed?

- ☐ Yes - please see below
- ☐ No

9.a.i. Could you tell us if your employment status is related in any way to your lupus.

10. What sort of paid employment do you do?

11. Do you work full or part-time

- ◯ Full Time
- ◯ Part time

11.a. Please tell us about your working pattern here.

12. Has your working pattern changed since you have been living with lupus? If so, please tell us how and why?

12.a. Could you tell us how you felt about these changes?

12.b. If your income has been reduced as a result of these changes, please indicate the extent of this change on the scale below, where 0 is low or no reduction and 10 is a significant reduction.

- ☐ 0
- ☐ 1
- ☐ 2
- ☐ 3
- ☐ 4
- ☐ 5
- ☐ 6
- ☐ 7
- ☐ 8

- [ ] 9
- [ ] 10

12.b.i. If you would like to tell us more about this, please do so here.

[ ]

13. How has your confidence in your abilty to maintain paid employment changed due to your lupus? Please indicate on the scale below where 0 is no change and 10 is significantly changed.

- [ ] 0
- [ ] 1
- [ ] 2
- [ ] 3
- [ ] 4
- [ ] 5
- [ ] 6
- [ ] 7
- [ ] 8
- [ ] 9
- [ ] 10

14. If you have never had paid employment, is this due to lupus?

- [ ] Yes
- [ ] No

14.a. If you would like to tell us more about this, please do so here.

15. Have you left paid employment because of your lupus? Please tick whichever applies.

- ☐ Medically retired
- ☐ Resigned
- ☐ Dismissed on capability grounds
- ☐ Chosen to retire

15.a. If you would like to tell us about the circumstances, please do so here.

16. Do you feel that your lupus has had any positive effects on your working life?

17. Are you claiming support / benefits as a result of your lupus?

- [ ] Yes
- [ ] No

17.a. If you would like to tell us more about this, please do so here.

18. If you are claiming support / benefits, how difficult or stressful do you find this? Please indicate the extent of this on the scale below, where 0 is low or no stress and 10 is a significant stress.

- [ ] 0
- [ ] 1
- [ ] 2
- [ ] 3
- [ ] 4
- [ ] 5
- [ ] 6
- [ ] 7
- [ ] 8
- [ ] 9
- [ ] 10

18.a. If you would like to tell us more about this, please do so here.

19. Has the impact of lupus on your working life affected your mental well-being? Please indicate the extent of this on the scale below, where 0 is low or no impact and 10 is significant impact.

- [ ] 0
- [ ] 1
- [ ] 2
- [ ] 3
- [ ] 4
- [ ] 5
- [ ] 6
- [ ] 7
- [ ] 8
- [ ] 9
- [ ] 10

19.a. If you would like to tell us more about this impact, please do so here.

20. Are you worried about the future becase of any impact that lupus has had on your ability to maintain paid employment? Please indicate the extent of your concerns on the scale below, where 0 is low or no worry and 10 is significant worry.

- [ ] 0
- [ ] 1
- [ ] 2
- [ ] 3
- [ ] 4

- [ ] 5
- [ ] 6
- [ ] 7
- [ ] 8
- [ ] 9
- [ ] 10

20.a. If you would like to tell us more about this, please do so here.

21. Do you think your employers understand that your condition can be very variable?

- [ ] Yes
- [ ] No

21.a. If you would like to tell us more about this, please do so here.

22. Do you think that your colleagues understand that your condition can be very variable?

- [ ] Yes
- [ ] No

22.a. If you would like to tell us more about this, please do so here.

23. If there is anything else you would like to tell us about your experience of lupus and paid employment please tell us here.

Page 4: Thank you

Thank you for taking the time to complete our survey. Your input is really important in helping us to understand the impact of lupus on working life.

Sara Booth, Liz Walker and Liz Price.
