# Supplementary material for: ‘When I asked for help and support it was not there’: current NHS employment practice and its impact on people with systemic lupus erythematosus
Source: Rheumatol Adv Pract. 2021 Mar 12;5(1):rkab019. doi: 10.1093/rap/rkab019 (PMC8058394; doi:10.1093/rap/rkab019)
Supplement: rkab019_Supplementary_Data [file rkab019_supplementary_data.zip › 20-142 Supplementary Data S2.pdf]

Box 1a
The direct negative effect of SLE on employment

P15: 45-54 clinician, SLE >15 years

'I have been bullied at work, I have faced discrimination trying to get new employment. I had to accept that I cannot consistently do things and for most jobs this is not acceptable, becoming significantly disabled at thirty is hard, giving up work has been essential but a disaster at the same time if that makes sense, this government hates the sick, it's degrading: they don't see why you need so many appts off. I used to work full time, then part time, then unemployed.'

P21: 35-44 clinician, SLE 6-10 years

Scored 9/10 financial impact of SLE, and loss of confidence and ability to continue working. 'I work 24 hours as that is a suitable amount that does not cause too much impact on health, I was working 30 but that was too much and I had to reduce as I couldn't manage. (feel) down (about these changes) as I want to financially do more to make ends meet but my body does not allow it. I am considering leaving work as I am not currently managing. Have highs and lows and feel also it affects my marriage as I'm not sure where this illness is going to take me and it is getting harder to cope.'

P29: 45-54 years, primary care admin, resigned from clinical role

'I now earn the minimum wage of £7.50 an hour when I used to earn £13 an hour: now work 4 hours a week when I used to work 12, sad. I used to work 40 hours a week and sleep in two nights. I had to give up the job I loved. I have to rely on my husband giving me money each month.'

P30: 45-54 years, non-clinical role, SLE for >15years, medically retired

'Yes I retired on I'll health due to chronic fatigue syndrome on direct relation to my lupus. I managed to have a full time job for 25 years by careful self-management of my symptoms and excellent support from Consultant and GP but in the end I feel my lupus "caught up with me" and I could no longer bounce back after illness.'

P53: 45-54 years, SLE 1-5 years, part time work 22.5 hours

'I was pursued for benefit fraud so HAD to look for employment as benefits stopped, worried. Caused too much stress being on benefits. I am struggling and do not expect to be able to continue with paid work for much longer. Not sure how we will manage money wise so that is what is making me continue. Consultant has told me to reduce hours or stop. If the benefit system was fairer then I wouldn't have to work.'

P66: 45-54 years, non-clinical role, working part-time 17 hours a week, claiming disability benefit

'I used to have a weekend job. I had to give that up unfortunately. I wasn't coping with my main job, I was off sick for a very long time so had to give up my second job. A drop in jobs is a drop in pay. I am worried. I don't know how long I have left to work. It's a struggle every day. I work in a surgery with brilliant dr's and rheumatology nurses. They are very supportive.'

P69: 55-64 years, managerial role, SLE 1-5 years

Full-time employment, scored 10/10 for impact of SLE on mental wellbeing and ability to continue working in future

'Loss of confidence and feelings of isolation due to working in a cubicle to protect me from extensive lighting. Concerned I may not be able to continue to work full time and the financial consequences of that. My immediate line manager also has lupus! She is very flexible with my timekeeping but I do need to do my hours.'

P72: 35-44 years, clinician, full-time, variable shifts, SLE <1 year

7/10 loss of confidence in maintaining paid employment

'Have become unable to work night shifts. Disappointed as I lose out on enhanced pay rates because of this, loss of enhanced rates for out of hours work.'

P81: 45-54 years, clinician, SLE 6-10 years

'…cannot do 12 hour day shifts anymore, too tiring. I only do nightshifts and sometimes still struggle, help majority of the time but still struggle sometimes.'

Box 1a
The direct negative effect of SLE on employment

P129: 35-44 years, locum clinician working part-time in short-term jobs after redundancy, SLE 11-15 years

'Work 4 to 5 days a week in late spring and summer and struggle to work in the autumn and winter, push myself to work 3 to 4 days a week because I have to work. Reduced working hours due to chronic fatigue, respiratory difficulties, painful muscular body, stress causes flares. I became very stressed, financially difficult. Unable to further myself professionally, brain fogs, and reduced finances. Made redundant, I don't tell employers about my health as this will influence their decisions to offer me work. More awareness and research is needed. Everyone is bombarded with information about cancer, little or nothing is known about lupus.'

P185: 35-44 years, senior managerial role in hospital, SLE >15 years

'Mon to Fri 9 – 5 plus on call: I have spent 16 weeks in hospital in last 5 years and am forced to work from home during recovery periods. Depressed, I had to leave front line clinical work as lupus has significantly compromised my immune system. Really hard decision to make. Sometimes wake up shattered. Fatigue is hard. Also depression. I hate letting lupus win so I tend to try and ignore it. As I am prone to sepsis which I have had 4 times in recent years this isn't always a good strategy. Every time I am admitted to hospital I get concerned over my sickness level. I only ever take time off when I am an inpatient so don't give myself proper recovery time.' *(understanding colleagues/managers)*

P193: 35-44 years, ward clinician now on benefits, SLE 1-5 years

'I lost my job due to my disease, *(feel)* bereft, I worked full time as a *(clinician)*. Now I am totally reliant on benefits. Dismissed on capability grounds, I was unable stay in work due to constant flare ups, I had to fight for 51 weeks to get the lowest level of PIP and I am now fighting to get ESA. I've had to sign on for job seekers' allowance even though I am so unwell. I have had to fight to obtain benefits which has had a hugely negative impact on my health and wellbeing. Giving up paid employment for the rest of my life isn't a viable option. My low income is hugely stressful.' *(understanding colleagues and employers)*

P199: 25-34 years, student ward clinician, SLE 11-15 years

'Currently at university studying ***(AHP)***. Previously, I had a part time job for 10 years. Originally told that I would not be able to work due to my lupus, however part time employment allowed me to have rest days to recover. I was previously employed part time as a bar manager, 4-6 hour shifts 5 days a week. Mostly evening work as mornings are more likely to be when my joints are swollen. Rest/recovery days became very important. And I feel I definitely require more time off than "normal" people due to hospital appointments and fluctuations in the state of my lupus. There have also been times, even when I was working, that I felt frustrated that I could not provide much income and have to rely on partner and family to help me out financially. I hope that through being honest and upfront about my condition I helped to highlight the existence of "invisible illness" to past employer and hopefully increase their understanding of the needs of people trying to work whilst managing a chronic health condition. Working part time allowed me to still have time for myself. I applied *(for benefits)* but was turned down due to "not being ill enough." My experience of the benefit process was incredibly negative. There are always people who are sceptical about the condition.'

Box 1b
The direct negative effect of SLE on employment

P230: 25-34 years, ward clinician, SLE 6-10 years

'I work 12 hour day shifts and 11 hour night shifts (full time). I have to work alternate day shifts so that I have at least one rest day after a shift. I can only cope with two night shifts together due to fatigue. Because my job is very physical I often have to delegate to other colleagues when my pain isn't under control. Because I'm unable to have shifts in a row it means I don't get the continuity that my colleagues get. When I have to delegate to my colleagues I feel that I'm not a team player and that I'm letting my colleagues down. We get unsociable pay for working night shifts. I can only cope with working a few night shifts a month which means my pay is less than if I was able to work more night shifts. I suffer with low mood and I'm very snappy and agitated when my pain is uncontrolled, I tend to be very negative. I'm worried that I will have to change the career I love because of the effect my lupus has on my physical and mental health. Because there is no physical representation of the disease my colleagues don't understand how it can effect me. People tend to thing I just haven't had enough sleep when I'm suffering with fatigue, I don't think lupus and its effects on daily life has been publicised enough. Flare ups can be very debilitating and I don't think people understand how much it effects the physical and mental health.'

P253: 35-44 years, community clinician, working part-time, freelance, SLE 11-15 years

'I am physically unable to work full time, I miss the money! I don't like not being able to do more… (I) resigned (because of my lupus) had to give up one practice I worked at to go part-time, my joints in my hands are affected and I need my hands to be good and strong to do my job so I worry how long I will be able to do it. I am always open and honest with the people I work with and luckily they all get it.'

P255: 55-64 years, ward clinician part-time, SLE 1-5 years

'I work as a bank *(clinician)* 2 days a week via an 'as needed contract' so no sick pay, I had to do reduced hours and was then finding this increasingly difficult…frustrated as the fatigue and recurrent infection left me tired and low in mood, could no longer manage a full-time post so retired and work 2 days for the service; chosen to retire (as) finding it increasingly difficult to manage work even on reduced hours. Post was a full time post and I could not manage this. I was lucky that I managed to keep my position as long as I did and that my manager did agree with occupational health that I needed to reduce my working hours. The extreme fatigue is something I had never experienced. If I go out at the weekend I am wiped out the next day. Recurrent infections have meant lots of antibiotics and this has led to a real feeling of feeling low in mood. No one seems to understand this. I constantly worry about *(the future)* as I am on my own. *(Employers understand)* to a certain extent my colleagues do not want a team member to be unreliable; it's difficult to explain to colleagues, I was lucky and I did have a supportive manager but I did not like to take time off away from work. I was always very proud of 'no illness at work.'

P268: 18-24 years, works in NHS, unspecified role, SLE 1-5 years

'Work full-time five days a week. Dragging yourself out of bed everyday to go to work when some days I'm really not sure it's even possible, is so hard, it makes me so fed up some days, some days I will lay and cry before getting up. I'm sure many people with lupus will relate in that, I just wish I could wake up one day and not ache anywhere or not feel rubbish, just to feel healthy would be a miracle!! I feel as a 21 year old I should still have energy, but I don't so how am I going to feel in 20 years time?! If I am honest I'm not too sure my employer even knows I suffer with lupus… Most of my colleagues don't have a clue what it is, and when I try and tell them, I get a feeling they think I'm lying or putting it on or just lazy. The struggles of full time employment and lupus are never ending.'

P281: 45-54 years, senior clinician hospital and community, SLE 11-15 years

'...work full time 2-4 12.5 hour shifts weekly, regular rotation onto nights. I work harder and take less time off to prove I am capable of my job. I find it extremely difficult to gain understanding as my condition cannot be seen.'

Box 1b
The direct negative effect of SLE on employment

P283: 45-54 years, medically retired from NHS as clinician/tutor, SLE >15 years

(With lupus, went) 'from full time clinician to DLT/Assessor, to not being able to work. Very disappointed and demeaned in no longer being able to find suitable work and felt a failure in not being able to function adequately in work, medically retired… I had left NHS work to pursue another direction feeling that the stress was too much and causing my ill health. …I was even advised that if my mood could improve I would find I no longer had SLE… There is still stigma as more people get to hear of lupus, it's like the lazy illness or hypochondria. It can be compared to MS but in the sense that MS is a real illness and lupus is just a made up fake illness.'

P331: 45-54 years, clinician working on bank post-medical retirement, SLE >15 years

'Work part-time on the *(named hospital)* bank short shifts 2-3 times weekly when able. I was a *(community clinician)* working full-time, managing a large caseload and a team of *(community clinicians). (Post-medical retirement)* I was really distressed at the time at having to leave, I understood that I was a burden to my colleagues and not there for my patients but it was also not my fault and I was having to give up a job I truly loved, I had found my niche and thrived on it…(the) ill-health pension, equated to losing half my monthly take home. Due to repeated flares mainly due to polyarthritis, fatigue and lupus lung conditions, I was off work for weeks and months at a time often returning to work too soon, soon I was off work more than I was at work. Management sent me to occupational health who helped me see I was never giving myself a chance to get better and the stress from my job was adding to my flares… my job is less physical now and that means I don't do things I know will stress me out. The loss of my career, friends, living with a chronic disease which changes with the wind, creating new and distressing symptoms, the loss of my home and lifestyle all combined to cause my mental health to suffer. Due to my age and continued flares I wonder how long I can go on for and the financial burden I will face through the years till I get my state pension. The fact that at the moment I am able to do any work is amazing and it helps my self-esteem no end. Working on the … bank …I am able to avoid ward work and do shifts like flu jab clinics, treatment room shifts, discharge liaison, addiction clinics so am very lucky, the bank is also aware of my limitations and offer me posts which are suitable to my health needs.'

P340: 35-44 years, ward clinician dismissed due to SLE, SLE 1-5 years

'I lost my job as a *(clinician)* due to my disease, I was dismissed from my *(clinician)* job due to having too much time off sick... *(I was)* devastated but I don't have long enough periods of being well to maintain a job. I am reliant on benefits. I had to fight for 51 weeks to be awarded PIP and now I am having to fight to be awarded ESA. I am having to fight for benefits and the DWP has found me fit for work despite my chronic illness.'

P353: 45-54 years, ex-ward clinician, SLE >15 years

'I'm unemployed due to lupus, was a former *(ward clinician)*. I had to give it up due to fatigue, brain fog, fatigue and pain. I have tried several jobs since but always had to give up as they make me poorly… it was heart-breaking to give up *(ward work)*, was very depressed initially... I'm on benefits ESA, and PIP. Dismissed on capability grounds. I had lots of treatment failures due to the fact it didn't get diagnosed for two years. Work gave me limited time to get better but I couldn't get the consultant to agree I would be able to go back to *(work)* so *(was)* retired on medical grounds. *(being on benefit)* its degrading and awful, you dread the brown envelope. *(Effect on my mental wellbeing)* it did very much initially but I have come to terms with it and have wonderful creative hobbies, I inherited enough money to buy a house so feel more secure. I was bullied in one job for taking time off, I would love to work and tried a variety of jobs, even tried to come up with *(manageable)* ideas for self-employment. I'm so disabled I now have a home help.'

Box 1c
The direct negative effect of SLE on employment

P371: 45-54 years, ex-clinician, dismissed on capability grounds, SLE 6-10 years

'Was a *(clinician)* for 20 plus years, I use to work shift work, now unable to work due to my low level functioning and unpredictability of how I may be. *(feel)* Devastated and is still difficult to accept. My life changed and coping with not only my physical health but the psychological, emotional and the financial was, and still is, difficult and exacerbated symptoms, then came more trauma of insecurity and everything I knew changed. Also dealing with drs and employers saying I needed to go back to work, it may loss my job *(unable to accommodate my needs and did not I feel listen to me)* No one in there right mind would choose this path and loss so much. Turned my family's life upside down. My income dropped hugely and I had to (when extremely unwell) had to fight for my ill health early retirement. I do not get long term /higher rate due to needing more time on treatment and more tests, it was said I would improve to be able to return to sedately *(sic)* employment… Dismissed on capability grounds… *(long passage on benefits system left out)*. HORRIFIC EXPERIENCE. Cruel and unbelievable ... *(effect of lupus on mental wellbeing)* Permanent grieving, live in fear and frustrating. From the illness itself and the impact it has on daily life, to accessing care and treatment and support, lack of awareness from other people and workplace to accessing financial help … *(worried about the future?)* Terribly worried, how I will keep my home and roof over how will I pay the bills, nothing is secure and everything I worked for could be taken away. I have made huge adjustments but yet in a few years will have to be interrogated again and justify claiming PIP. I will have to find new evidence, I will have to try and get letters supporting how it effects me. I have a go who is at a loss most of the time and am sent from dr to dr and from dept to dept feeling and always someone else's problem. He *(sic)* feels I need care coordinated due to the complex needs but no one doing this. Referred to dept over an hour and half away and offered treatment support ... to far to keep traveling as exhausting, mobility probs and financial price. *(colleagues attitudes)* I did not feel that I was taken seriously when I told them. Especially whilst being investigated. The diagnosing and tests take time often many, many months then to get the help and treatment due to waiting lists. *(managers understood variable condition)* I am able to manage life and my symptoms are better if I am able to pace and rest as and when I need too. Sometime my illness gives me no warning and I have no choice. Please believe us when we say how we feel and please make work place adjustments. My partner gave up work to care for me, claiming carer allowance. We struggled to make ends meet. He had since tried to get part time work, all zero hours, absolute nightmare system. He had worked all his life, and nearly had a breakdown with all the benefit system and the zero hour contracts. The system fails thoughts *(those)* who genuinely cannot work or care for loved ones.'

P380: 45-54 years, senior clinical manager, SLE 11-15 years

'Retired on ill health at 42 from NHS. Now work as an *(agency researcher)* to allow me to pick and choose when I'm able to work, Part time, four to eight nights a month, I was a *(senior clinical director)* in the NHS. Working 60 plus hours a week. Retired on ill health. Now work 60 hours a month, when I'm feeling well enough. I was very angry. I worked hard to achieve my career aims, now I feel very fortunate just to be able to work at all. My retiring salary was 68K. I now earn with pensions and work around 35K.' *(both employers and colleagues understood variability of condition)*

Box 2a
Lack of employment support from NHS

P44: 55-64 years, retired clinician, SLE 11-15 years

'Chosen to retire…too much time taken from work resulted in threats from my employer (NHS) of loss of pension rights and dismissal…management treated me with no compassion, no understanding and made an awful situation far worse…'

P76: 45-54 years, clinician, part-time work, SLE 11-15 years

'I am meant to work 27 hours a week, but recently some weeks I can work 11.5 hours, and another week 46 hours! I have complained but my employer is uncooperative. I reduced my hours from 30 hours to 27 so that my working pattern suited me better, but as per my previous answer my employer, an NHS hospital, disregards this completely. It has made a big impact financially and physically and emotionally. My employer, NHS trust, is very uncooperative and uncaring.'

P93: 35-44 years, clinician ward, FT work, SLE >15 years

'Variable shifts to cover 24 hours period, I have worked more nights during times of heightened photosensitivity. I have found the mental stresses of dealing with my employer very damaging. My employer has stated openly that my lupus is an excuse to pick and choose what shifts I work, did not even consider reasonable changes to my working hours during a flare and I have had to take significant periods of sick leave rather than work reduced hours. This has lead to my employer following disciplinary proceedings against me for sickness absences. I have repeatedly asked for adjustments to prevent this happening, accepting that my income will drop but my employer will not consider this as an option and feels I am unreasonable to request any such changes. My employer would still have had access to my skills and mentoring of junior staff if I had been able to reduce hours but they felt it was more fitting that I remove myself from the environment altogether when having a flare as it "creates too much paperwork." I have found the mental stresses of dealing with my employer very damaging.'

P95: 45-54 years, community clinician, just dismissed on capability grounds, SLE 6-10 years

'Reduced to 30hrs sometimes have to start later due to muscle pain, joint pain, particularly in mornings. I have recently had my employment medically terminated due to ongoing health issues. Dismissed on capability grounds. Lupus combined with anxiety and depression have led to medical termination after 32 years (clinical work). I'm out of a well-paid job to possibly go on a very small pension.'

P104: 35-44 years, part-time work, SLE 1-5 years

'I work 8hrs shift only and not more than 5 shifts consecutively. I felt better, as I was struggling with the 12 hrs shift. The 12 hrs shift was causing my lupus to flare a lot more. I work weekdays and not weekends, so I do not get the enhancement, so basically I get a basic salary only. It worries and stressed me a lot because I'm scared I will lose my job. As I work with NHS and they are really strict with the sickness level and they do not care if you are living with any chronic disease(s). I'm worried I will lose my job and unable to get another job. As you can have a flare up without any warning.'

P135: 35-44 years, now living on benefits, previous NHS clinician, SLE >15 years

'Unable to work, employers lack of understanding of condition. Frustrated as I still have so much to offer to society. Previously sleeping in my car during my lunch hour during a flare. Requested reduced hours and refused. Dismissed on capability grounds, Because lupus cannot be 'seen' (fatigue, joint pain, headaches) people don't take you seriously. Only when one side of my face collapsed did people show concern! I try to stay positive but am constantly worried about my health situation and being able to live (pay bills) Huge worry! I want to be able to work but am terrified to get back into the situation when homelessness was a real possibility. I would need to retrain as this condition is made worse if I could get employment in the sectors I previously

worked in. Retraining means (you are 'fit for work' even though you may not be). I worked for the NHS and desperately relied upon my break to sleep in my car. When I was discovered sleeping in my car I was send to occupational health who said my hours should be reduced. My line manager made this difficult and I ended up on SSP, and so the journey to ESA began. The frustrating thing is I had been managing my condition successfully for years (having a nap in unpaid time, my lunch hour!). Because you cannot see joint pain, fatigue etc you are not considered to be suffering from a 'real' illness. Until lupus is understood diagnosis will continue to take too long, complications will become too advanced (In my case a tx) and employability being occasional instead of the norm. I worked for the NHS and then as a teacher and in both positions lupus was not known about.'

P144: 25-34 years, hospital clinician, SLE 6-10 yrs

'Unable to do extra shifts due to fatigue and frequently sick. I am very rarely not fatigued or tired. Poor quality of life as always too tired to do anything. Won't be able to keep up this pace without getting sick *(employers)* don't really get it. *(colleagues)* don't really know about it it's an awkward convo.'

P171: 35-44 years, hospital clinician, SLE 11-16 years

'I was full time and doing on call before I became ill. I also had the opportunity of overtime on weekends. As a result of my sickness record I was forced into reducing my hours. I was told if I didn't I could risk losing my job *(I was)* Devastated. There has been a huge financial impact on me but also it was damaging from a psychological point of view. My confidence had suffered as a result of my illness and the attitude of my employers particularly in relation to my ability to do my job was damaging…all in all I lose about £1000 per month as a result. I am a single woman with a mortgage and bills so you can imagine the impact this has had on my life *(the effect on my mental health)* Confidence. Stress. Worried about keeping my job. I have also been passed over for opportunities and promotions in the past as a result of my illness. I never know how long I will be able to maintain my current hours for. Also the NHS has a strict sickness policy that does not allow for chronic long term relapsing conditions so I always have the threat of a disciplinary hanging over me. Because of this I have ended up having to use my annual leave to recuperate rather than take a sick day.' *(managers and employers do not understand that it can be variable)*

P172: 35-44 years, hospital clinician, SLE 6-10 years

'I can't work as much the pain the lights … managers are crap tried sacking me for having lupus. Not happy with managers trying sacking me for lupus and that's the NHS for ya.'

P178: 45-54 years, hospital clinician, SLE >15 years

'I am only able to work three days a week. I work 10 hour shifts at work. I get up at 530am and am in work to start at 8am. I work to 6pm get home at 630pm get dinner chill, and bed at 9pm. Three day only two together then a rest day then another work day *(changes to work pattern are)* Awful, that I needed to get them, as it was too much doing any more days.'

P195: 23-34 years, ward clinician, SLE 1-5 years

' ...*(working pattern)* More time off work sick, need for flexitime, banking overtime to use as time off. Like a poor employee, loss of self- esteem, unable to pace effectively. Even though I work for the NHS there is low understanding of my condition and minimal acceptance/tolerance for the reasonable accommodations I need...*(employers)*… Seen as complaining, "always tired".' *(by colleagues)*

Box 2b
Lack of employment support from NHS

P196: 45-54 years, ward clinician, about to retire/be dismissed, SLE 1-5 years

'I work 2 shifts a week 19 hours. Since diagnosed with lupus have gone from full time to part time. Unable to manage pain, stiffness and fatigue to try and cope I have gradually reduced hours to part time even though financially it's a struggle *(feel about these changes)* Distressed, depressed and anxious. Nobody understands what my body is like and how difficult life is. I am now on half the wage I was, it's a big drop but I cannot cope with the demanding work and standing all day. I am being managed and may have to take medical retirement before I am managed out of my position due to levels of sickness. *(benefits)* I have just been to a terrible tribunal hearing where I have been awarded PIP at the lower rate. It took over 14 months from the start and I still have not received any payment.'

P201: 45-54 years, community clinician dismissed on capability grounds, SLE 1-6 years

'I lost my job as a *(clinician)* with local health trust I was very upset to lose my job and income. They were meant to deploy me but didn't, dismissed on capability grounds, I receive employment support, non-working group, lost lot of sight due to lupus, I can feel lost and no purpose, due to significant sight loss, fatigue and other lupus symptoms. I know I couldn't commit to work, but feel it's another loss.' *(no support from employers)*

P239: 35-44 years, ward clinician, SLE 11-15 years

'*(work)* full-time, 9-5 Monday-Friday, 9-5 Monday-Friday, I changed from shift work, I felt regular hours would be better, but it's still same workload. Found the NHS discriminatory and was told by middle management I should work in a lupus clinic, as everyone that has a disease should work in that department. My issues resolved immediately when a legal letter was sent to NHS Managers.'

P263: 45-54 years, left NHS, SLE 1-5 years

'Self-employed in retail, full time, allows more flexibility. Was working full time in NHS, found full time work tiring. I requested to reduce hours due to fatigue and mother had been diagnosed with breast cancer, which also impacted on mental health. NHS declined so I resigned, depressing. Was an upper band 6 specialist (clinician). So had a massive reduction in my income. As I'm now self-employed, it's more stressful in that I cannot take time off due to sickness and if I do I lose money. Also constant fatigue has an impact *(9 for worry about future employment)* Need to be well and fit enough you to earn money to meet day to day living requirements. ...when I asked for help and support it was not there.'

P290: 35-44 years, medically retired senior clinician, SLE >15 years

'I was forced to medically retire from my occupation as *(senior clinician)* 18 months ago following long periods of unpredictable SLE related symptoms. I breached the sickness policy. *(no current employment)* When in employment I reduced hours from full time to 30 hours and then 25, 20 & 18 due to progressive fatigue & prolonged absences. I stopped night duty, reduced working hours, alternative roles not well accepted by management. Reduced shift duration from 12 to 6 hours. *(I felt)* Stressed. Burden to management. Sent to Occupational health at every opportunity waiting specialist investigations. Felt I would lose my job. Felt a failure. I was the main earner in my household. My self-employed husband's work is irregular. We almost lost our home. Our parents paid our bills, mortgage & continue to support us. My husband now has 3 jobs. My medical pension is less than half my earnings. I have accepted I may never work in paid employment again, however I hope for remission & change in circumstances to return to something in the future. My employers were waiting for me to breach the sickness policy again. I should have been redeployed at diagnosis but that failed to meet my needs. Fear of losing respect, lifelong friendships, my job, my income was hidden from everyone until my world crashed in around me. However it's only money. I will never have the quality of life & health I had. There is no cure. My condition continues to flare.'

Box 2b
Lack of employment support from NHS

P293: 45-54 years, ex-HR worker NHS, SLE >15years

'Work full time often late, in HR, Try to work from home a couple of days a month. Exhausted all the time, go to work then come home and go to bed. Love working from home, no long drive, resigned *(from NHS)*, Left HR work in NHS because of extreme exhaustion, Sjogren's and related depression. DWP treat people terribly. Concerned that I might not be able to support my family if I get ill. I have not told my current employer as it is still career limiting, I know – I'm in HR. Do not discuss.' *(with colleagues)*

P296: 55-64 years, ex-mental health clinician, SLE >15 years

'Dismissed from employment due to lupus, worked full-time until dismissed, I have a private pension which I could draw as I was over 55. Dismissed on capability grounds. New sickness policy introduced which was more stringent and this caused a loss of stress. The pressure of losing income and not being able to replace it is horrendous. I was made to feel like I was a fraudster, trying to claim something which I was not entitled to. The fear of losing your job and income when you have a flare up consumed my life at times. My manager put constant pressure on me to retire which I didn't want to do. This caused low mood and great anxiety. I couldn't sleep for worry at times which had a negative impact upon mood and ability to manage day to day. I am afraid to apply for employment now because I fear that I would not be able to maintain employment. Luckily my husband is in employment and supports me but without him I would struggle financially. When employed I gave my employers a booklet produced by Lupus UK aimed at educating employers as to the effects of Lupus and how they could support mean my employment. They refused to reduce my caseload when unwell and told me that if I was at work then I was presumed fit for work. When well Lupus did not affect my ability to undertake my role and duties. When unwell there was little or no provision to support me to undertake my job.'

Box 3
Changes in employment that supported better health

P65: 35-44 years, full-time work, with supportive colleagues and manager, SLE >15 years

'Yes. I've changed jobs. I have previously worked in the NHS, lots over overtime, some unpaid. More regular hours now than before. I assessed what I'd really li(k)e to do, a less stressful job, I have a better job so it pays more. I've always worked no matter how I felt, I never wanted to be different from everyone else. I also studied for my degree while working and worked while studying accountancy. I resigned *(from previous NHS job)*. Too many hours effected my lupus plus I wanted to study again.'

P106: 25-34 years, NHS *(clinician)*, working full-time flexibly, outside normal pattern for career, SLE 1-5 years

'…taking a year out of training due to SLE being problematic, essentially work full time. However my working pattern is pretty good. My week is split into 10 sessions, 4 clinical (working on a ward or in clinic), 4 teaching (medical student lectures or bedside teaching), 2 sessions of personal time to work on my PGCert in education. And then I work one evening a week (5pm-9:30pm) in the medical receiving unit and then either a Saturday or a Sunday every 3-4 weeks. *(working pattern has changed with lupus)* Very much so. I have taken a year out of training as my health wasn't great and I would definitely not be able to cope with a full rota with nights and runs of longs days. *(it's)* Sad. However it needs to be done. And I would rather work at a reduced rate than not work at all. I find work very therapeutic and it keeps me occupied. My mental wellbeing suffers when I am not at work. *(My employers)* I work in the NHS and have to deal with patients with SLE so although they *(NHS)* aren't the best, they have a vague grasp and I can just point them in the direction of their text books.'

P113: 55-64 years, left NHS, previously admin in NHS, SLE >15 years

'…indirectly – I can manage well by working from home. Previously I worked self-employed at a Consultant's home running his private practice – which again made coping with lupus easier, I am not sure how well I would cope if I had to go out to work, as opposed to working from home, I manage most of the time but sometimes it is difficult, however, I have to work to make a living and being self-employed I have no sick pay.'

P123: 45-54 years, clinician, SLE 1-5 years

'I have more flexibility to work on different days and times depending on how I feel. Less stressful than feeling pressured to go in when unwell. Try and keep to core hours of about 20 a week but this varies depending on how I am feeling. Sometimes do more one week to catch up if less done previous week. Noticeable reduction in income was a shock initially. Overall very pleased to go part time and accept I just can't do what others my age do because of my health issue. I am at an age where I can relax more now and I am married. Had I remained single with mortgage I would have been very worried about my finances and lifestyle.'

P356: 55-64 years, auditor *(private)* company, SLE 6-10 years

'Work part-time. I have a flexible working plan I am allowed to work from home but am expected to go to the office once a week however if I'm unwell am excused. As long as I complete my 6 hours my employers are flexible when I start and finish. *(I'm)* Relieved *(at changes in work pattern)* Medically retired by previous employers *(claiming)* PIP. The process was stressful and demeaning. I now suffer from social anxiety. I have a lack of confidence despite have more experience than most colleagues. I feel 'left behind' as cannot apply for promotion as I am part time. I have had periods of sickness and although my employers are supportive I am aware they are not generally tolerant of extended sick periods. My immediate line manager is particularly understanding plus we are a health care company and I am a medical professional. I lost my career and very well paid employment with previous company and although I am grateful I can carry out an IT based role with my current company I feel I lost a successful career.'

Box 3
Changes in employment that supported better health

P164: 45-54 years, community admin, SLE 6-10 years

'Previously worked full time in management, relief *(to change)* 45K to 10K, (I) Resigned, Employer not accommodating of illness, stressful job, idiot boss. Walked away from job to look after my health. Best thing I did. Love my current job and life! Probably would still be in full time job I disliked if it weren't for lupus, Significant IMPROVEMENT in my mental wellbeing by changing job and work life balance. I work in a (community hub) so they have good insight into condition. I've had one day of illness in 5 years so they know I'm not a work shy, idle malingerer. Previous employer not so understanding. Boss tried to mark me down at appraisal for losing my speech (due to migraine) during a teleconference. Wouldn't give me part time hours.'

P176: 45-54 years, admin in community, full-time, 5 days a week, SLE >15 years

'Used to work 12 hours shifts, it became too much...' *(colleagues and managers understanding)*

Box 4
Supportive interventions from NHS system enabling continuing employment

P72: 35-44 years, clinician hospital, SLE <1 year

'My job is very demanding physically and mentally and i dont believe i would be able to do this until retirement age. I am currently trying to further my education so as to put me in a position where i can go into management or education both of which would be less clinical and therefore less physically demanding, working for the NHS my employer does understand my condition and to a certain extent are supportive but i still feel much more could be done.'

P215: 55-64 years, ward clinician, SLE 1-6 years

'…3 x 8 hour shifts, full time night shifts too much, became very unwell so on three 8 hour shifts now, Felt pretty useless that couldn't carry out my job properly and that colleagues had to stand in for me. I am on part time wages now and still ill most of the time, but cannot afford to retire or work less shifts. My line manager is very good, working in NHS does help. But some of my co-workers don't understand. Some new co- workers think I am just lazy, which is very frustrating. I think on the whole I am lucky with my employment. Working within the NHS they do understand my condition.'

P249: 35-44, non-clinical specialist, SLE >15 years

'…work full-time, weekdays 9-5 (ish) hours and length of day vary depending on workload regularly. No longer clinical, due to lupus symptoms. Have increased hours due to financial need, significantly increased commute hours to work, very negative impact through increased hours and commute, but unavoidable as income required, lack of clinical work has positively affected lupus, worry over ability to maintain work and income as a single parent with no family to support if ill or unable to work, have had significant flares causing time off work – worry may one day not be able to work as i am, but have to, to support my children and myself. Know i should really work less hours because of impact on my health, but cannot afford not to. I have excellent line managers who are nurses and fully support me and also allow me to adapt my working when required – i.e. work at home, not travel. reduce my workload, my immediate colleagues, *(understand variability)* but not the rest of my organisation. I have been very lucky with 2 supportive NHS employers. I would not be working now or not to this level without their support and my health would certainly have suffered without their help and adaptions. They have been excellent.'

P296: 35-44 years, ward clinician, SLE 6-10 years

'(Work) 80% of full-time, day time only, (reduced because of lupus) stopped out of hours work, works well for me, I was reviewed by Occupational health after I became unwell and the adjustments to my work have made an enormous difference. Having a day off mid-week means I only work two days in a row and helps me manage the fatigue. I am fortunate that my job is well paid and I can afford to work reduced hours. So far I have not had any issues about taking time off but my employer uses the Bradford factor which discriminates against people with chronic illness who need to take multiple short periods of sick leave…' *(employers and colleagues understand condition is variable)*

Box 5
Fear about future employment

P110: 45-54 years, hospital clinician, SLE >15 years

'As I get older I worry I may get more complications as a result of lupus and the medications I take to treat it. This could make working full time difficult.'

P137: 45-54 years, hospital clinician, SLE >15 years

'Struggled with fatigue mid-week so have a day off every 2 weeks which gives me a recovery day to let me balance home life and work *(change pattern was)* My choice so happy Lost confidence in my ability to retain information – often fatigued so memory can let me down. Close colleagues *(understanding)* do but feel I need to explain that I have SLE to others when I am having a flare or days when I need to go home early because of the fatigue or generalised joint pain. *(I)* would like to cut hours but worry about what might happen in the future if I have periods of absence due to my lupus flares so working full-time just now while I can manage.'

P163: 55-64 years, retired, SLE >15 years

'I had a serious episode of renal lupus and was advised to retire. I had worked as a medical secretary and was struggling to work with all my symptoms. Lupus has meant that I never really enjoyed my work as it was always a physical struggle and I never achieved my potential. I constantly worry my benefits will be removed even though I am a genuine case. I also feel a bit ashamed because I realise I am a burden to other tax payers. Having to leave work aged 32 has undermined my confidence and isolated me. I am fortunate to have a husband with a modest pension, but I still feel vulnerable financially as I get older. When I worked I tried desperately to conceal any symptoms which looking back was a mistake. However, many of the symptoms I had, such as extreme fatigue, aches, mild fever etc, would have been hard to explain because I always looked well.'

P185: 25-34 years, ward clinician, SLE 1-5 years

'I work two long days a week 7:30am to 8:30pm, one early shift 7:30am to 3pm and one late shift 12:30pm to 8:30pm. I often end up missing my breaks and staying later than my shifts due to short staffing. I find the shifts physically exhausting and that has an impact on my mental health. I am constantly worrying about the impact of the stress of the job on has on my body, as historically stress has caused me to have quite severe lupus flares and hospitalised with sepsis on more than one occasion. I worry that if I am off sick too much due to my lupus employers will dismiss me. However I know when I begin to flare that if I dont get on top of it I will end in hospital. Most of the senior staff at the hospital do not understand what lupus is, again *(colleagues)* don't know what lupus is.'

P257: 18-24 years, SLE 1-5 years

'I am at university doing *(ward specialty)* and I also work in care as a health care support worker, Bank Shifts usually 12 hours then I have placement at university working in hospitals which is 3-4 12 hour shifts a week for 9 weeks 2-3 times a year. I've tried to continue going as much as I can. It's just difficult at times with the fatigue and you feel so tired at times it doesn't matter how much you sleep and I was scared I made a mistake due to this. I will become a fully qualified *(clinician)* and I didn't know I'd get this when i started university course. But working in a hospital I'm more open to people with infections and I'm more immune to getting infections *(sic)* which scares me. *(employers)* Not a lot of people know about lupus and most people I've spoken to about it are negative.'

Box 6
Feelings of shame, guilt and being a burden to family and colleagues

P118: 45-54 years, admin staff, SLE 1-6 years

(working pattern changed) 'For the worse. Had to take shifts on board and full time to get jobs. *(it's)* Exhausting, ok to start but get tired as week goes on. *(I've)* Resigned, I finish tomorrow as unable to cope with stress, depression and maintaining concentration in my job. Being asked to do extra jobs and overloading my brain, (my) Determination to try and do the best I can under the circumstances. Try to be as productive as colleagues. Due to this I have been pushed to the limit mentally and physically and had to resign as feel not firing on all cylinders and feel a failure, I think the only way out is to stop working in 2 years when luckily my husband retires but I will only be 50. No way I could work until 67. I tend not to disclose (SLE) in case I don't get the job and then find it difficult to approach the subject of explaining this all. I also have Sjogren's. They probably just end up thinking I'm a stress head and dithery old lady. I mention it to colleagues but not management. One manager was quite caring about it due to time off work and changing me to 4 days a week.'

P274: 35-44 years, admin staff, SLE 6-10 years

'...I went from full time working in a gym to having to go part time as an administrator but financially I needed to go back to full time as I have 2 kids and was struggling money wise but now I'm full time I have had flares up and become unwell as it's too much. I used to be so active and had the energy and enthusiasm to work full time but now I can't do long days and I feel drained but financially it is so hard. I feeling like a failure to my family like I'm not trying even though I am and I just can't get anywhere in my life as I feel I can't no longer work full time as I get ill. I have been dismissed on capability grounds. I went for a job position that I was currently in as temp staff for over a year but as soon as they found out I had lupus I did not get the job fully due to capability. My lupus is an invisible disease to the world there is no intervention help like were I struggle to work full time due to my long term illness but will never get any help from benefits to subsidies for this. I'm always depressed and stressed out I'm physically and mentally drained because you sit there pretending that your well when your ill is draining because people do not understand the disease and treat you like you are lying.'

P262: 45-54 years, AHP, SLE 1-5 years

'Work full-time, have only done this for 9 months, it's exhausting. I am anxious that my colleagues feel I am given an easier time or seen as less reliable. I have used all my leave to attend medical appointments.' *(colleagues and employers do not understand variability)*

P240: 18-24 years old, SLE 1-5 years

'I was medically resigned from my health care job a year into having lupus. I no longer work. *(feel)* guilty, useless, ashamed, I have gone from £1200 to £500 a month. Medically resigned from a senior health care assistant a year into having lupus. The job was too physically and emotionally draining. I claim ESA… *(10/10 stress applying for benefits)* Very stressful time because lupus cannot be seen. I was frightened of being rejected because I look fine. Often feel low about myself. And ashamed I do not make my own money at a young age. Worried that it will be difficult to find a job when I'm ready to return to work due to the large gap in my CV. *(with colleagues and managers)* Again, it cannot be seen and there's little general knowledge about it.'
